# Proximal-end bias from *in-vitro* reconstituted nucleosomes and the result on downstream data analysis

**DOI:** 10.1371/journal.pone.0258737

**Published:** 2021-10-21

**Authors:** David A. Bates, Charles E. Bates, Andrew S. Earl, Colin Skousen, Ashley N. Fetbrandt, Jordon Ritchie, Paul M. Bodily, Steven M. Johnson

**Affiliations:** 1 Department of Microbiology and Molecular Biology, Brigham Young University, Provo, Utah, United States of America; 2 Qubit Software LLC, Spanish Fork, Utah, United States of America; 3 Computer Science Department, Idaho State University, Pocatello, Idaho, United States of America; Universität Stuttgart, GERMANY

## Abstract

The most basic level of eukaryotic gene regulation is the presence or absence of nucleosomes on DNA regulatory elements. In an effort to elucidate *in vivo* nucleosome patterns, *in vitro* studies are frequently used. *In vitro*, short DNA fragments are more favorable for nucleosome formation, increasing the likelihood of nucleosome occupancy. This may in part result from the fact that nucleosomes prefer to form on the terminal ends of linear DNA. This phenomenon has the potential to bias *in vitro* reconstituted nucleosomes and skew results. If the ends of DNA fragments are known, the reads falling close to the ends are typically discarded. In this study we confirm the phenomenon of end bias of *in vitro* nucleosomes. We describe a method in which nearly identical libraries, with different known ends, are used to recover nucleosomes which form towards the terminal ends of fragmented DNA. Finally, we illustrate that although nucleosomes prefer to form on DNA ends, it does not appear to skew results or the interpretation thereof.

## Introduction

Chromatin is the combination of DNA and DNA-associated proteins. A histone octamer, made up of eight histone proteins, serves as the first means of DNA compaction and organization [1]. DNA wraps around a histone octamer ~1.7 times to form a nucleosome [2, 3]. In a nucleosome, there are two types of DNA positioning: rotational and translational [4, 5]. Rotational positioning is the way the DNA double helix interacts with the histone proteins as it turns; translational positioning is where the nucleosome forms laterally along the piece of DNA. In most contexts, the phrase “position” refers to the nucleosome translational position.

As the first means of DNA compaction and organization, where nucleosomes form has a significant role in basic cell processes such as transcription, DNA replication, and DNA repair; and by extension has a role in more elaborate cell states such as differentiation and cancer. With such far-reaching consequences, understanding what positions nucleosomes becomes paramount. Thus, reconstituting nucleosomes *in vitro* becomes a powerful tool in understanding the patterns and changes of nucleosome positioning.

A commonly used method for determining high-nucleosome-affinity DNA sequences is through the use of *in vitro* nucleosome reconstitutions. Whole-genome applications of this method begin with isolation of protein free genomic DNA followed by generation of smaller DNA fragments primarily through sonic shearing or restriction enzyme digestion. Recombinant or isolated histone octamers and DNA fragments are then added together in high-salt solution in a stoichiometric ratio such that on average a single nucleosome will form on each individual DNA fragment. The salts in the solution are then dialyzed away, allowing the formation of nucleosomes [6, 7]. Nucleosome positions from the *in vitro* reconstituted assemblies can be compared to their *in vivo* genomic equivalents, allowing for the identification of not only high-nucleosome-affinity sequences determined exclusively by intrinsic DNA sequences, but also the amount of *in vivo* remodeling that occurs within individual cell or tissue types. Such an approach was used by Locke et al. to demonstrate the extent of nucleosome remodeling that occurs *in vivo* on the *Caenorhabditis elegans* (*C*. *elegans*) genome [8].

While *in vitro* nucleosome reconstitutions provide valuable information, the technique contains at least one inherent bias that must be overcome to fully use the derived data. It has been demonstrated that DNA-fragment ends can influence nucleosome formation, encouraging end-proximal nucleosome formation relative to the remainder of the DNA fragment [9, 10]. This preference, given names such as proximal end-bias, fragment end-bias, terminal end-bias, end effect, and end bias, can introduce a major hurdle when attempting to identify high-nucleosome-affinity DNA sequences. Because of this major bias, in any *in vitro* nucleosome reconstitution experiment, it becomes impossible to determine if *in vitro* nucleosome (hereafter referred to as invitrosome) [11] formation is due to end bias or an actual affinity for the underlying nucleotide sequence.

When reconstituting nucleosomes *in vitro*, there are four major methods of reconstitution that have been used in recent years. First, reconstituting on whole chromosomes [12]. Second, reconstituting on large (5kb and larger) linear or circular fragments of DNA [13–15]. Third, reconstituting on genomic DNA that has been sheared [8]. Fourth, reconstituting on short artificially synthesized DNA sequences [16, 17]. Each approach has advantages and limitations. Reconstituting on whole chromosomes or large DNA fragments requires low levels of protein, otherwise precipitation occurs; therefore, what few nucleosomes do form will be highly attracted to the sequence upon which they form. Using shorter, sheared DNA can utilize higher protein levels and thus results in more extensive levels of nucleosome formation; however, as discussed above, the nucleosomes that form have an unusual propensity to form near the ends of the DNA fragments. While shearing DNA via sonication or ultra-sonication may have appeal due to the appearance of randomness (i.e., the acoustic energy being indiscriminate in the breaking of the phosphodiester bonds of the DNA), evidence suggests that it may not be as random as initially thought [18–24]. DNA sheared, or rather cut, by enzymatic digestion of specific DNA sequences may not be as popular as other methods, but the DNA ends can be determined with relative ease and accounted for in the downstream analyses if type II restriction enzymes are used. Finally, DNA that has been artificially synthesized is limited to the number of different molecules that are synthesized and may not adequately represent a genome that utilizes nucleosomes for chromatin formation. This last method is typically used for chromatin remodeling assays and frequently incorporates a nucleosome positioning sequence such as the Widom 601 sequence [25].

Nucleosome positioning is not random. The extent to which nucleosomes positioned *in vivo* due to ATP-dependent chromatin remodelers vs. positioning by DNA sequence is still debated. Positioning by DNA sequence can be measured by free energy, which is determined by intrinsic DNA features that form more stable nucleosomes based on the energy required to form a particular nucleosome. Chromatin remodelers, alternatively, can force or move nucleosomes onto DNA sequences that are much less favorable. Invitrosomes, in contrast, are positioned primarily by DNA sequence when chromatin remodelers are absent from the reaction.

Much research has been conducted to investigate how, and to what extent, nucleosome positioning occurs due to DNA sequence by examining trends in the underlying nucleosome DNA sequences. It has been found, both *in vivo* and *in vitro*, that the DNA surrounding the dyad (or midpoint of the nucleosome DNA) is rich in G/C nucleotides [26, 27], and G/C dinucleotides are overrepresented with a ~10 bp periodicity where the major groove of the DNA contacts the globular domain of the histone octamer [8, 27–32]. In contrast, the ends of nucleosomal DNA and regions flanking a nucleosome (linker DNA) are over represented in A/T nucleotides [8, 26, 33, 34], with a depletion of A/T nucleotides at the dyad [8, 27, 33], as well as an over representation of ~10 bp periodicity of A/T dinucleotides within the nucleosomal DNA [13, 25, 27, 28, 30–36] where the minor groove of the DNA contacts the globular domain of the histone octamer. Nucleosome depletion has been observed at promoters [13, 31, 34, 36–40], transcription termination regions [13, 34] (although this might be caused by proximity of termination sites to the promoter of downstream genes, as demonstrated in yeast [41]), certain short DNA tandem repeats and motifs [40, 42, 43], DNA replication origins [34, 44, 45], and Z-form DNA [46, 47]. Nucleosome enrichment has been observed at certain short DNA tandem repeats and motifs [40, 48–50].

It has been observed *in vivo* that nucleosome depletion occurs at homopolymeric stretches of A/T nucleotides [34, 40, 51, 52], transcription factor sites [36–38, 40], intergenic regions [34, 37, 52], 5’ UTRs [37], pseudoexons [53], strong mRNA splice sites [53], telomeres [34], and CpG islands [26, 54]. Nucleosome enrichment has been observed at exons [53, 55] and weak mRNA splice sites [53]. When comparing the underlying DNA sequences associated with nucleosomes, it has been shown that both enrichments and depletions between *in vivo* and *in vitro* datasets have a degree of positive correlation [8, 12, 13, 27]. However, some observed preferences may be species specific as demonstrated by differences between *S*. *pombe* and *S*. *cerevisiae*, such as enrichment of A/T nucleotides around the dyad and a depletion in A/T nucleotides near linker DNA in *S*. *pombe* compared to *S*. *cerevisiae* [56].

With these preferences/biases of nucleosomes known, two conclusions can be made. One, that there are rotational positioning biases (rotational biases), and two, that there are translational positioning biases (translational biases), with both types based on the underlying DNA sequence. The periodicity of G/C or A/T dinucleotides in nucleosomal sequences would be considered rotational biases [13]. Nucleosome depletion at Z-form DNA would be considered a translational bias, especially considering certain sequences are more prone to form Z-form DNA *in vivo* and *in vitro* [46, 47]. An additional type of translational bias that is not based on the underlying DNA sequence, the focus of this work, is end bias. And while other types of biases occur naturally *in vivo*, end bias is purely an experimental artifact. One research group observed that invitrosomes preferentially moved to the ends of linear DNA when exposed to elevated temperatures but tended to stay where they initially formed when kept on ice [10, 57]. Another group noticed a markedly higher percentage of invitrosomes that formed within 200 bp of known ends on linear DNA even when kept at cooler temperatures [8]. Finally, others saw that over 75% of invitrosomes were formed on the ends of their DNA fragments [9].

Here using previously published invitrosome data sets as well as our own invitrosome libraries, we answer the question of how extensive end bias is in *in vitro* nucleosome experiments. We use a high-throughput data analysis metric for evaluating end bias, followed by a computational method to identify and reassess potentially end biased invitrosomes. We also demonstrate that the effect of end bias on subsequent data analysis appears to be minimal, thus reassuring that conclusions from previous studies using *in vitro* nucleosome reconstitution to elucidate nucleosome DNA preferences were not skewed by end biases.

## Materials and methods

### Genome and libraries used for analysis

The *C*. *elegans* genome build WS190 was modified to replace the repetitive regions with “N” bases using the program RepeatMasker [58]. The WS190 version was chosen to be consistent with the build that was used for the Locke et al. analysis [8]. The four nucleosome sequence libraries used in this study were the 9.5 million read *RsaI* and 5.3 million read *HincII* libraries used in the Locke [8] analysis. These raw 36-bp single-end read libraries were trimmed to 25 bp before use to be the same read length as our other two libraries. A 25-bp single-end library of 8.0 million reads was generated *in silico* [59] (hereafter referred to as ART after the name of the program used to generate it) from the *C*. *elegans* genome. The ART program allowed us to simulate Illumina reads with the appropriate amount of sequencing errors an Illumina platform would have. The fourth and final library was a 25-bp single-end library of 20.3 million reads from invitrosomes assembled on ultra-sonicated *C*. *elegans* genomic DNA hereafter referred to US.

### Ultra-sonicated invitrosome library prep

Ultra-sonicated *C*. *elegans* genomic DNA was prepared as previously described [8], except that whole genomic DNA was ultra-sonicated (Covaris M220) to an average size of 700 bp, run on an agarose gel, size selected for fragments between 600–800 bp, excised and extracted. Recombinant histone proteins were purified as described [7], and invitrosomes were reconstituted by salt dialysis at a 1:1 molar ratio of DNA and histone octamer as previously described [8]. Isolation of invitrosome core DNA fragments was as described [8], followed by Illumina library prep (Illumina TruSeq DNA Library Prep Kit) and 25-bp paired-end sequencing on an Illumina HiSeq 2500. For end bias analysis only the forward reads fastq file was used.

### Library mapping and dyad calling

All libraries were mapped to the modified WS190 reference genome using Bowtie2 [60] on the Galaxy platform [61], with 7.8 million, 4.7 million, 8.5 million, and 18.7 million reads mapping from the ART, *HincII*, *RsaI*, and US libraries, respectively. Parameters for all programs used were set to default except as described in S1 Table. A bespoke Java program was used to calculate the location of all invitrosomes by computing the center or dyad of each invitrosome based on the end-sequence alignment and orientation. Invitrosome dyad positions were used to recover 147nt invitrosome sequences for k-mer analyses, whereas end-sequence alignment positions were used in end bias calculations.

### Invitrosome positions and end bias calculations

We wanted to measure end bias in two different ways: first, by measuring the ratio of the raw reads on the end of DNA fragments compared to all aligned reads, and second, by calculating the invitrosome coverage on the ends of DNA fragments. For the first approach we wanted to be precisely specific and only measure when invitrosomes started (i.e., the first base of a read). Aligned BAM files were modified [62] so each read in the library retained only the genomic position of the first base of the mapped read. Thus, each read now represented only the start position of an invitrosome when we used these modified alignments. To measure the ratio of the raw reads on DNA fragment ends, a custom Python 3.6 program (see availability section) was used to determine if invitrosome start positions, as listed in the modified BAM files, were located on the fragment ends at position 1 up through position 73. These numbers were divided by total aligned start positions, and subsequently graphed. End bias could only be analyzed for the *RsaI* and *HincII* invitrosome libraries, as they are the only invitrosome libraries where the DNA fragments used in the reconstitution had defined ends.

For the second analysis, we used the bamCoverage tool in the deepTools2 [63] suite. In this analysis, occupancy and coverage are synonymous. Using the modified BAM files, bamCoverage calculated invitrosome start coverage by first normalizing to reads per kilobase million (RPKM) to help compensate for the differing sequencing depths of the different libraries. Then the program calculated coverage. A custom Python 3.6 program was used to retrieve start coverage values from bamCoverage output at fragment ends at position 1 up through position 73. Then a mean start coverage value was calculated across the entire 73 bp region. The start coverage value for each position was divided by the mean start coverage value, and then graphed.

Two of the libraries used were also used in Locke et al. [8]. The two libraries not from the Locke analysis used for this comparison were: the ART library, generated *in silico* [59] to represent a purely random non-nucleosome sample of reads from a complex genome, and the sonicated US library, an invitrosome library generated by nucleosome reconstitution using ultra-sonicated DNA to represent a “pseudo-random” DNA nucleosome library as described above. Additionally, the genomic locations of *RsaI* and *HincII* cutsites (i.e., fragment ends) had been previously generated by Locke et al. and then filtered to exclude any *RsaI* or *HincII* restriction fragments smaller than 147 bp long for these respective analyses.

### Recovery method

The first step in our recovery approach was to use an additional custom Java program to extrapolate the location of each invitrosome by computing the center or dyad of each invitrosome based on the Bowtie2 alignment, calculate the invitrosome ends, and then separate invitrosomes into the categories of “suspect”, “passed”, and “innercut”. Suspect invitrosomes fall within the user-defined distance of cutsite (end) locations. Passed invitrosomes fall outside the user-defined distance of cutsite locations. Innercut reads represent invitrosomes that contain a cutsite within the nucleosomal DNA itself, which if the DNA were fully digested by the given restriction enzyme, would not be able to form invitrosomes.

**Fig 1:**
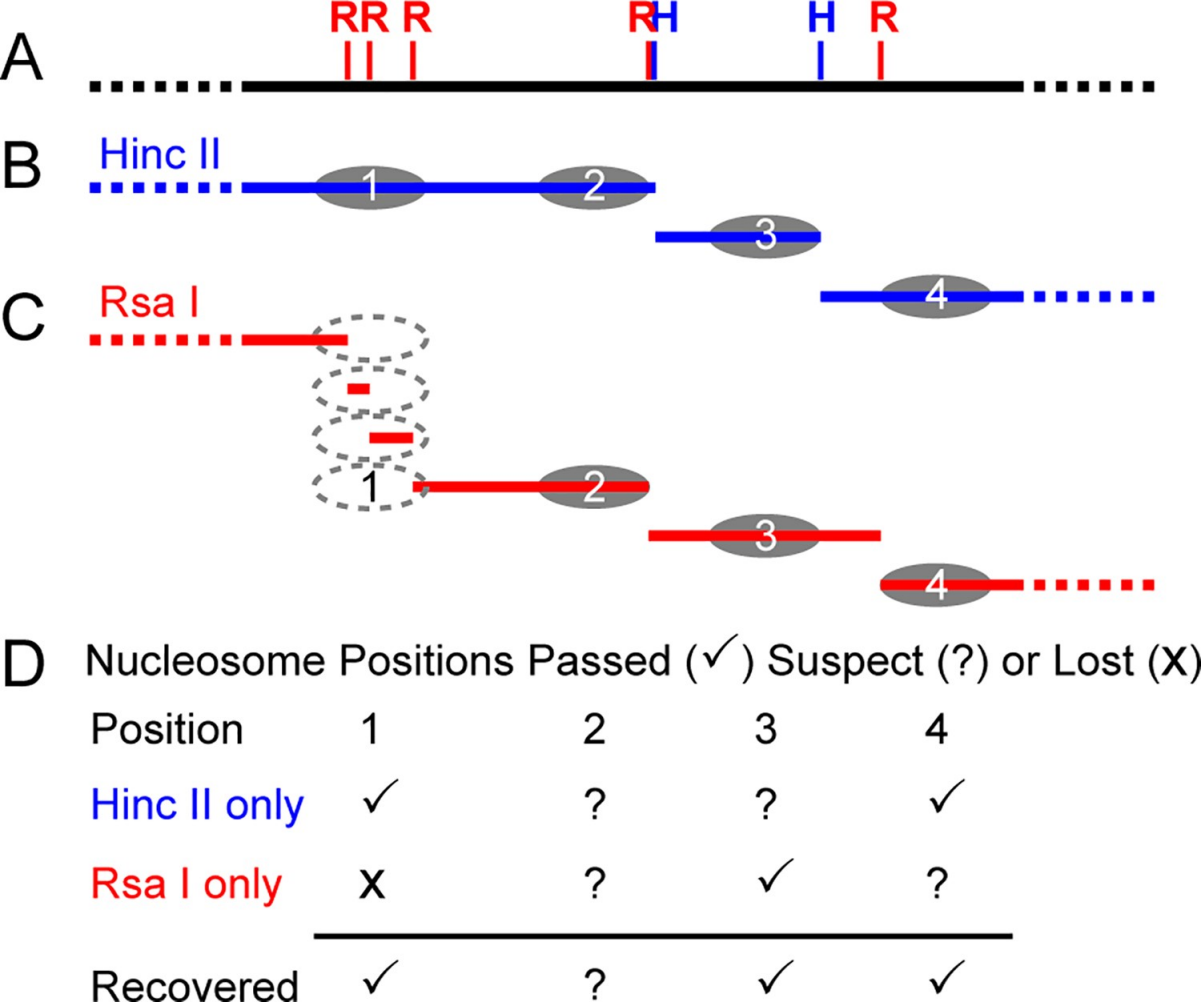
Visual depiction of the initial classification and re-classification method. A) Represents a section of genomic DNA with *RsaI* and *HincII* cutsites. B) Representation of the initial fragments of genomic DNA (blue), digested with *HincII* and subsequent reconstituted nucleosomes. C) Representation of the initial fragments of genomic DNA (red), digested with *RsaI* and subsequent reconstituted nucleosomes. D) Invitrosome initial classification and subsequent re-classification when compared to the alternate library. A passed or non-suspect invitrosome from one dataset (such as *HincII*) can recover or save all suspect or lost invitrosomes in the same location in the other dataset (such as *RsaI*) as demonstrated by position 1 and position 4 nucleosomes.

Next, suspect invitrosomes were recovered by comparison to the alternate experiment’s set of passed invitrosomes. Suspect *RsaI* invitrosomes were compared to passed *HincII* invitrosomes and innercut *HincII* invitrosomes. Reciprocally, suspect *HincII* invitrosomes were compared to passed *RsaI* invitrosomes and innercut *RsaI* invitrosomes. Suspect invitrosomes that sit at the same position as passed invitrosomes or innercut invitrosomes in the alternate library were re-classified as “recovered” or saved. A visual depiction of the initial classification and re-classification process can be seen in Fig 1. Invitrosomes that do not receive this new classification were considered biased invitrosomes. This resulted in a set of passed, recovered, biased, and innercut invitrosomes for each restriction enzyme library. Based on their classification, all invitrosomes were sorted into sub-libraries: Raw Library (passed + recovered + biased + innercut), Pre-Recovery (passed), and Post-Recovery (passed + recovered + innercut).

### Invitrosome nucleotide composition comparison

The DNA sequences from the four categories of invitrosomes (passed + recovered + biased + innercut) were used to calculate the nucleotide frequency of the underlying DNA sequences associated with each type of invitrosome. The same custom Java program used to find the invitrosome dyad, was used to pull out the invitrosome DNA sequence for each invitrosome in each category, and then calculate the rate of the various 4-mer combinations in the invitrosome DNA for each category in a position-based manner. Pearson and Spearman’s correlation coefficients were calculated for each 4-mer ratio in the *RsaI* or *HincII* libraries compared to the 4-mer ratios of the ART and US libraries at each position along the 147 base pairs of the invitrosome DNA.

## Results and discussion

### End bias occurs near the ends of DNA fragments

The propensity for *in vitro* reconstituted nucleosomes (invitrosomes) to favor formation on the end of DNA fragments has been observed by previous studies [9, 10]. These studies looking at end bias occurring *in vitro*, were based on low throughput data, did not quantify the amount of end biased nucleosome positioning, and did not analyze if this end bias affects down-stream analyses.

To confirm and quantify the end bias phenomenon we developed a novel method to calculate invitrosome occupancy at and around known DNA fragment ends. Using experimentally and *in silico* derived invitrosome libraries from the *C*. *elegans* genome, we quantified the number of invitrosomes that sit at the end of defined DNA fragments at base-pair resolution. We used four invitrosome libraries: two previously published [8] invitrosome libraries derived from *C*. *elegans* genomic DNA fragmented via enzymatic digestion (*RsaI* library, and *HincII* library), one invitrosome library derived from *C*. *elegans* genomic DNA fragmented via ultra-sonication (US library), and one library generated *in silico* [59] from the *C*. *elegans* genome (ART library). We aligned all four libraries to the *C*. *elegans* WS190 reference genome in which all repetitive elements had been masked with N’s.

**Fig 2:**
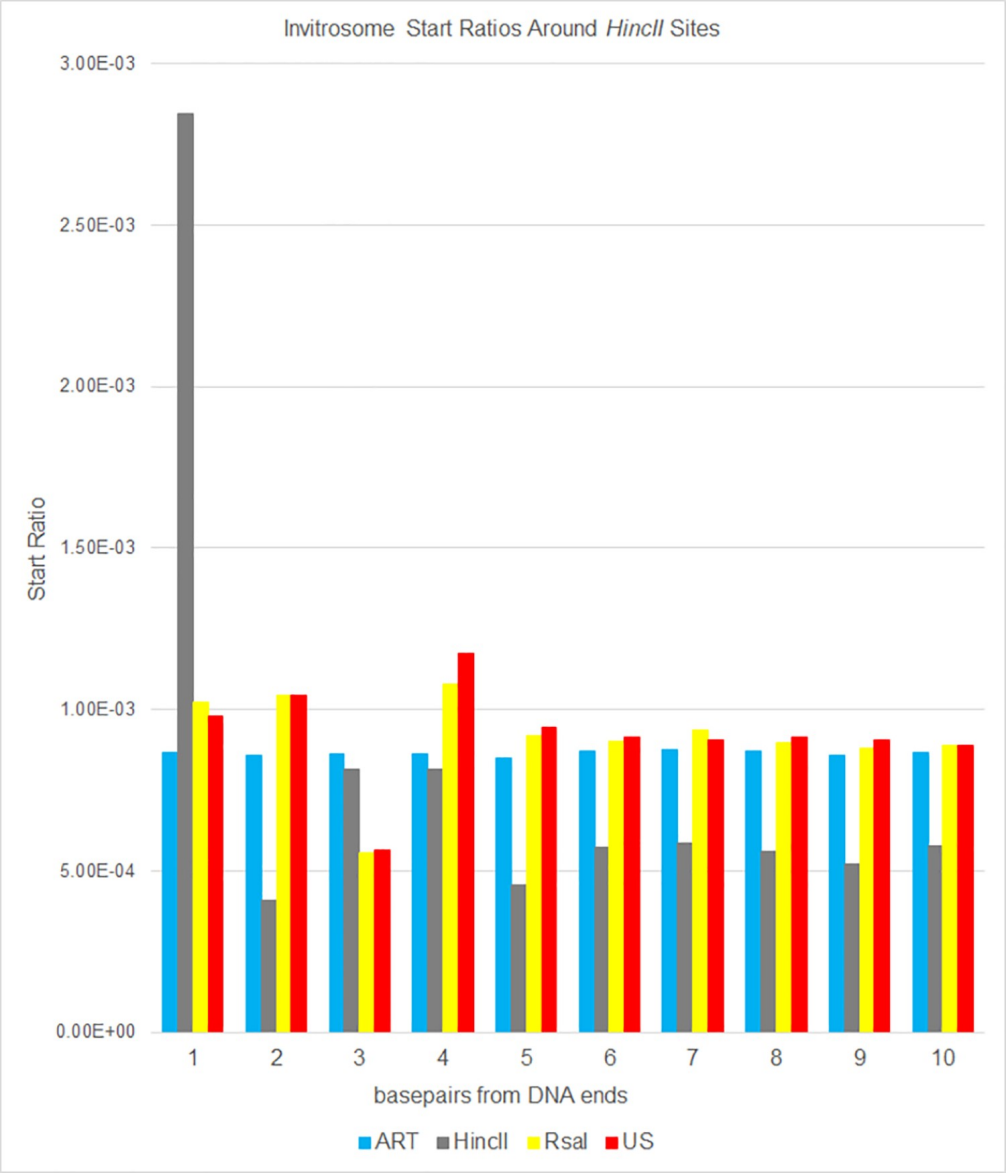
Ratios of invitrosome starts around *HincII* restriction sites in the genome. Ratios were calculated by taking all invitrosome starts at a given position and dividing that number by all invitrosome starts across the genome for each of the four invitrosome data sets (ART, HincII, RsaI and US) individually. Positions 1 through 10 on the x-axis are relative to *HincII* cut sites across the genome.

**Fig 3:**
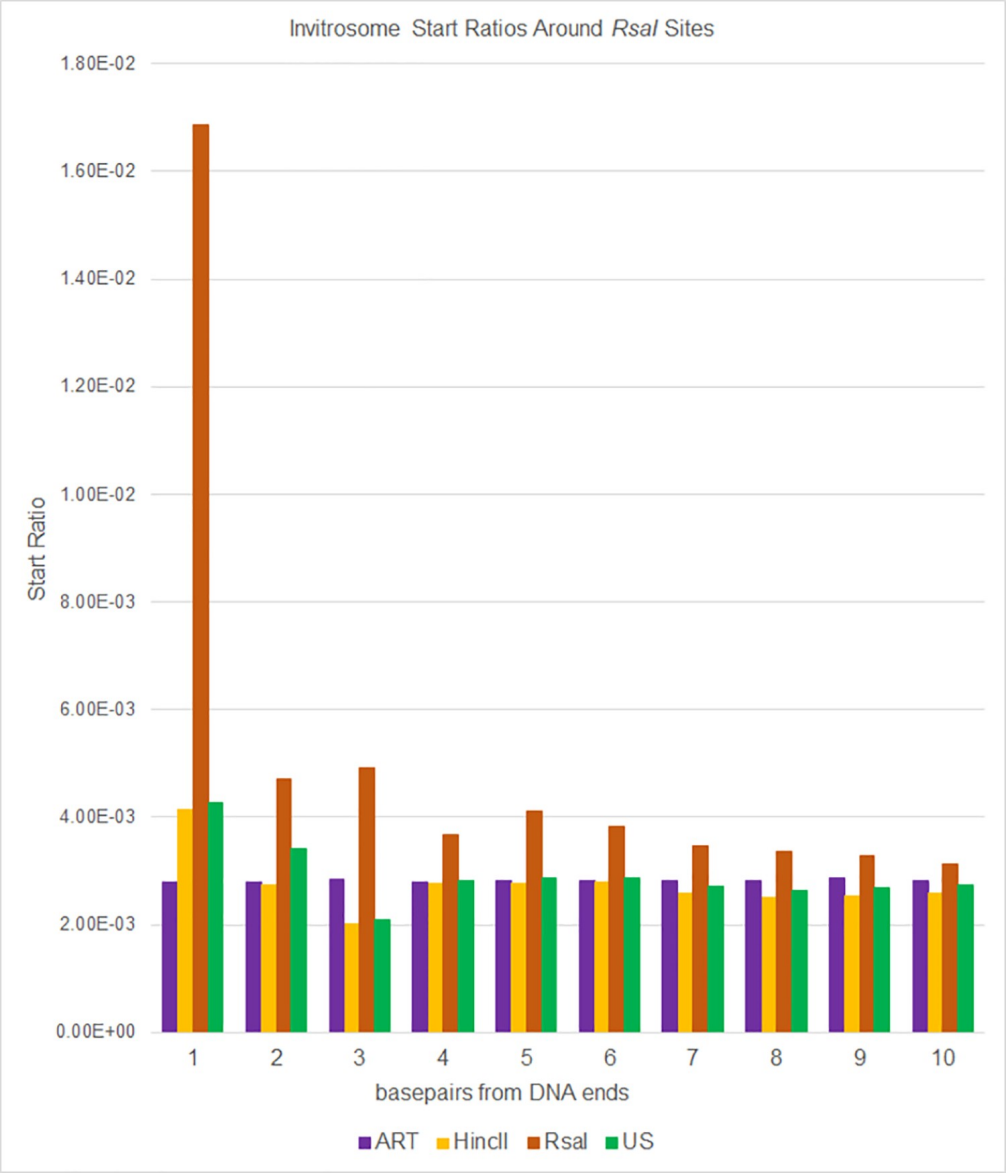
Ratios of invitrosome starts around *RsaI* restriction sites in the genome. Ratios were calculated by taking all invitrosome starts at a given position and dividing that number by all invitrosome starts across the genome for each of the four invitrosome data sets (ART, HincII, RsaI and US) individually. Positions 1 through 10 on the x-axis are relative to *RsaI* cut sites across the genome.

**Fig 4:**
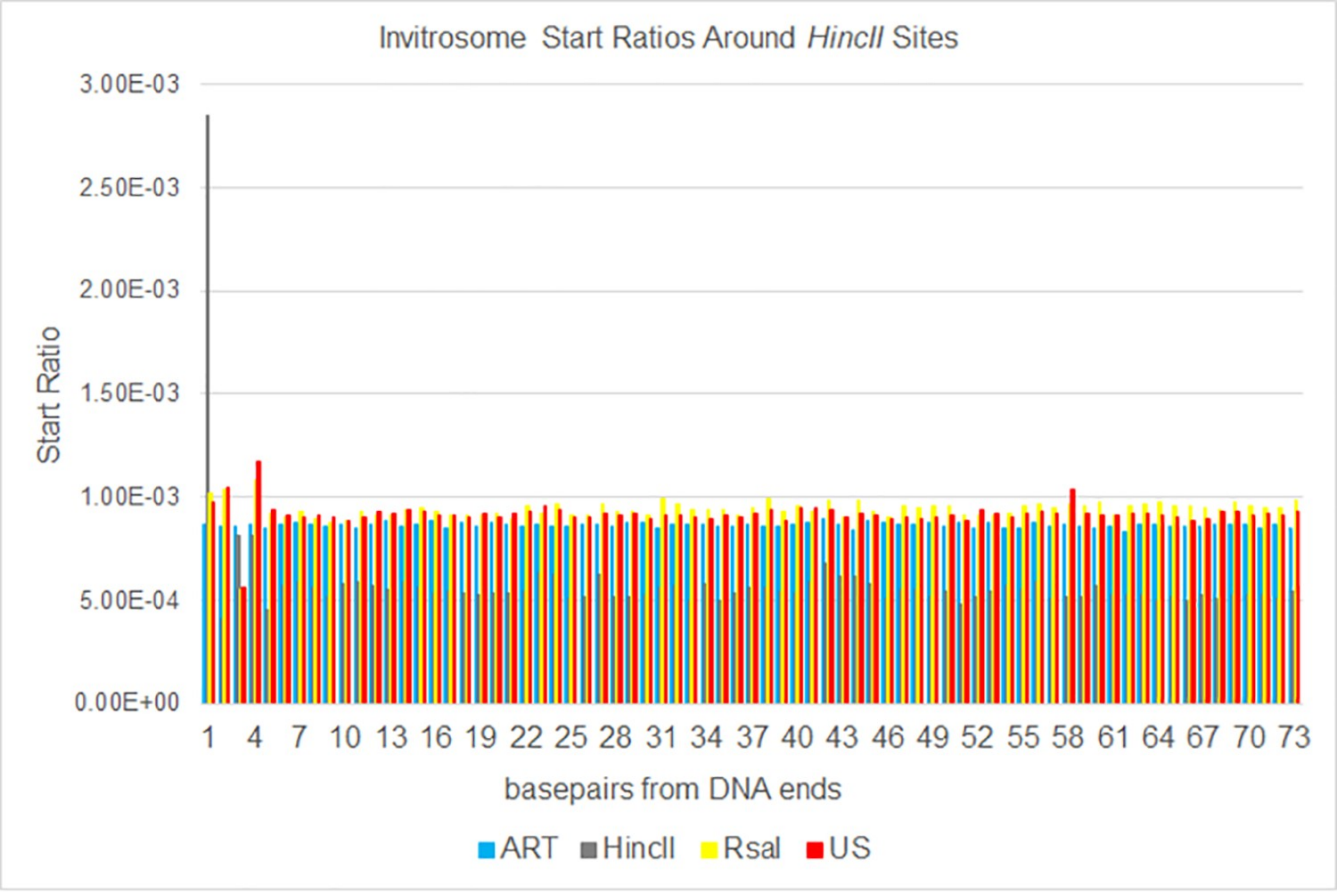
Expanded Fig 2. The same data as shown in Fig 2 expanded out to 73 bases from genomic *HincII* cut sites.

**Fig 5:**
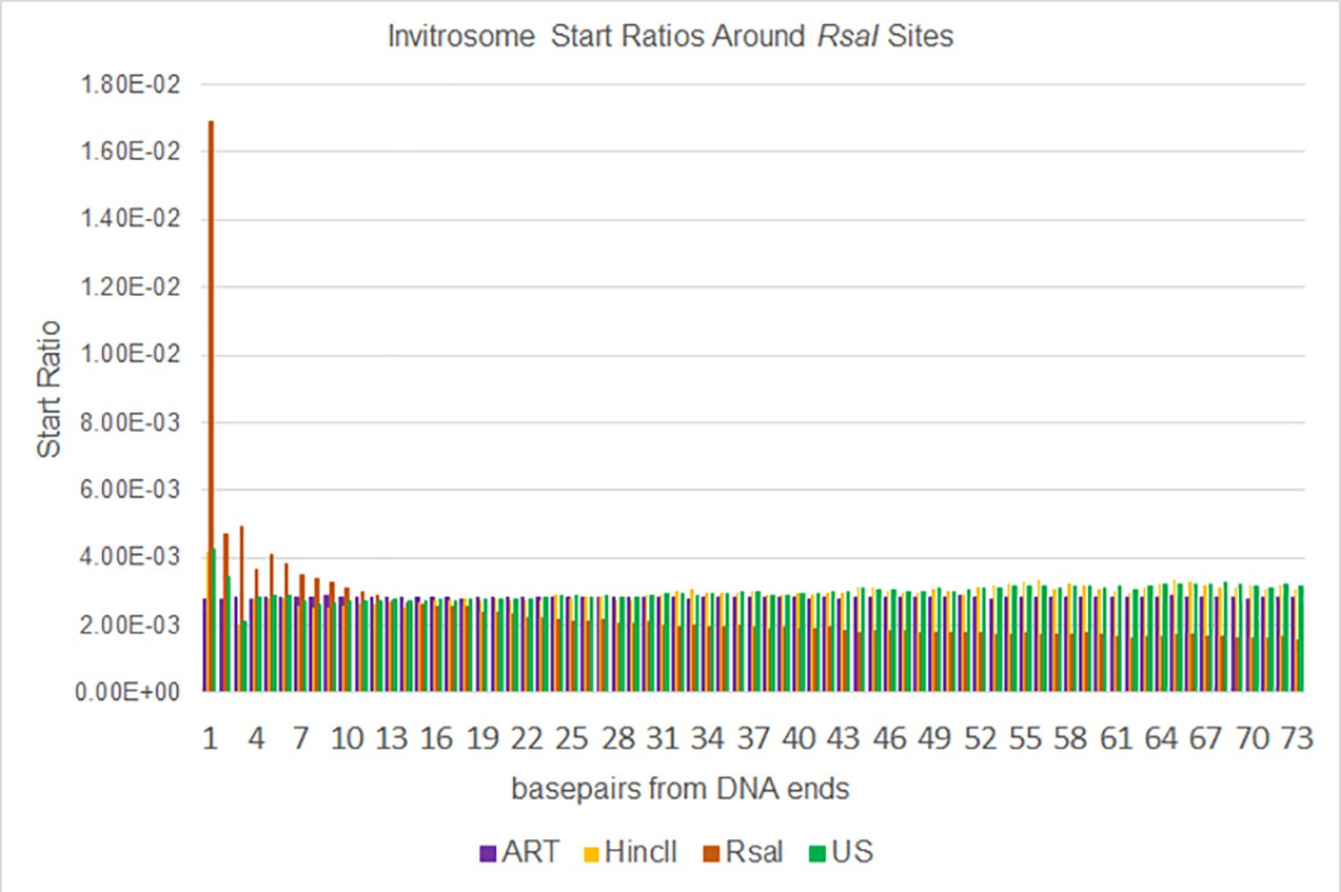
Expanded Fig 3. The same data as shown in Fig 3 expanded out to 73 bases from genomic *RsaI* cut sites.

Next, we calculated invitrosome start ratios at each base pair from position 1 (the precise end of the DNA fragments) out to position 73 (half a nucleosome length) by taking the number of invitrosome starts at a given position divided by the total number of invitrosome starts across the whole genome. We then graphed these ratios out to 10 bp and out to 73 bp from the DNA fragment ends. The data show that there is an unusually high start ratio right on the end of the DNA (i.e., position 1), that quickly approaches a baseline value within a handful of base pairs in from the end (Figs 2 and 3). Aside from a handful of bases past position 1, the ratio typically fluctuates around a baseline for the entire distance out to 73 bp (Figs 4 and 5). Interestingly, there is a divergence when comparing *HincII* and *RsaI* data around their own respective cutsites. The *HincII* data have a pronounced dip at position 2, rises for positions 3 & 4, dips at position 5, and then appears to fluctuate around a baseline ratio. The *RsaI* has a dip at position 2, rises at position 3, dips at position 4, rises at position 5, and then appears to approach an asymptote ratio. These differences could be explained by either the different sequencing depths of the libraries, or by the different cutsite sequences and restriction site lengths, or both. However, the very high ratio values at position 1 and the higher-than-average ratio values near the ends suggest that invitrosomes tend to form on or near fragment ends. This is further supported by analysis of the *RsaI* dataset using *HincII* cutsites and the *HincII* dataset using *RsaI* cutsites (reciprocal enzyme control), which both showed no such increased ratio at position 1. It is important to note that the ART values did not fluctuate much. Also, US values did not fluctuate much unless there was also a similar pattern in the *HincII* and/or *RsaI* reciprocal enzyme control values, possibly indicating that these positions contain very moderate invitrosome positioning or repelling sequence motifs in the DNA.

Seguin-Orlando et al. [24] investigated whether or not the methodology of Illumina sequencing introduced biases into high throughput sequencing data. They observed that the ends of fragmented DNA that was Illumina sequenced showed a depletion of thymine bases by ~11% and a parallel enrichment of adenine and guanine bases. Of note, the aforementioned enriched bases are present on the ends of *RsaI* and *HincII* digested DNA; however, we conclude that this sequencing bias is not the major contributor to the nucleosome enrichment we see on the very ends of DNA fragments. This is due mainly to the magnitude off the enrichment we observe. *HincII* digested DNA ends could begin with adenine or guanine on the 5’ end; and we observe an invitrosome enrichment of 278% at *HincII* DNA sites in our *HincII* data when compared to the next highest library value at position 1 in Figs 2 and 4. *RsaI* digested DNA 5’ ends begin with adenine; and we observe an enrichment of 396% when compared to the next highest library at position 1 in Figs 3 and 5. Thus our observed invitrosome enrichment on the ends of *RsaI*-and *HincII*- digested DNA is more than an order of magnitude greater than what could be explained by Illumina sequencing bias. Additionally, the much larger percent enrichment of invitrosomes for the *RsaI* library at position 1 compared to *HincII* library is likely due to the different recognition sites of the two enzymes. *RsaI* recognition sites are 4 bp, whereas *HincII* recognition sites are 6 bp. As such, *RsaI* sites occur more frequently in the genome, and digestion generates more DNA ends; thus, the availability of DNA ends is greater and provides more opportunity for preferential nucleosome formation on DNA ends with *RsaI* digested DNA when compared to *HincII*.

**Fig 6:**
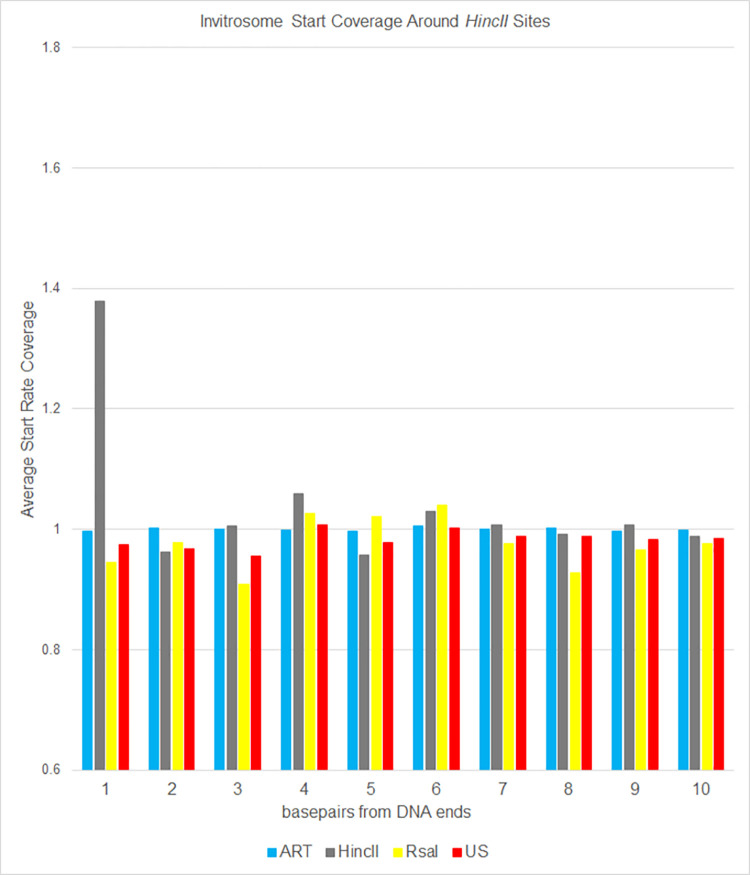
*HincII* occupancy of positions. Average occupancy (or coverage) of invitrosome starts around *HincII* restriction sites in the genome for each of the four invitrosome data sets (ART, HincII, RsaI and US). Positions 1 through 10 on the x-axis are relative to *HincII* cut sites across the genome. A value of 1 indicates occupancy at an average rate across the genome. Higher than 1 indicates above average occupancy.

**Fig 7:**
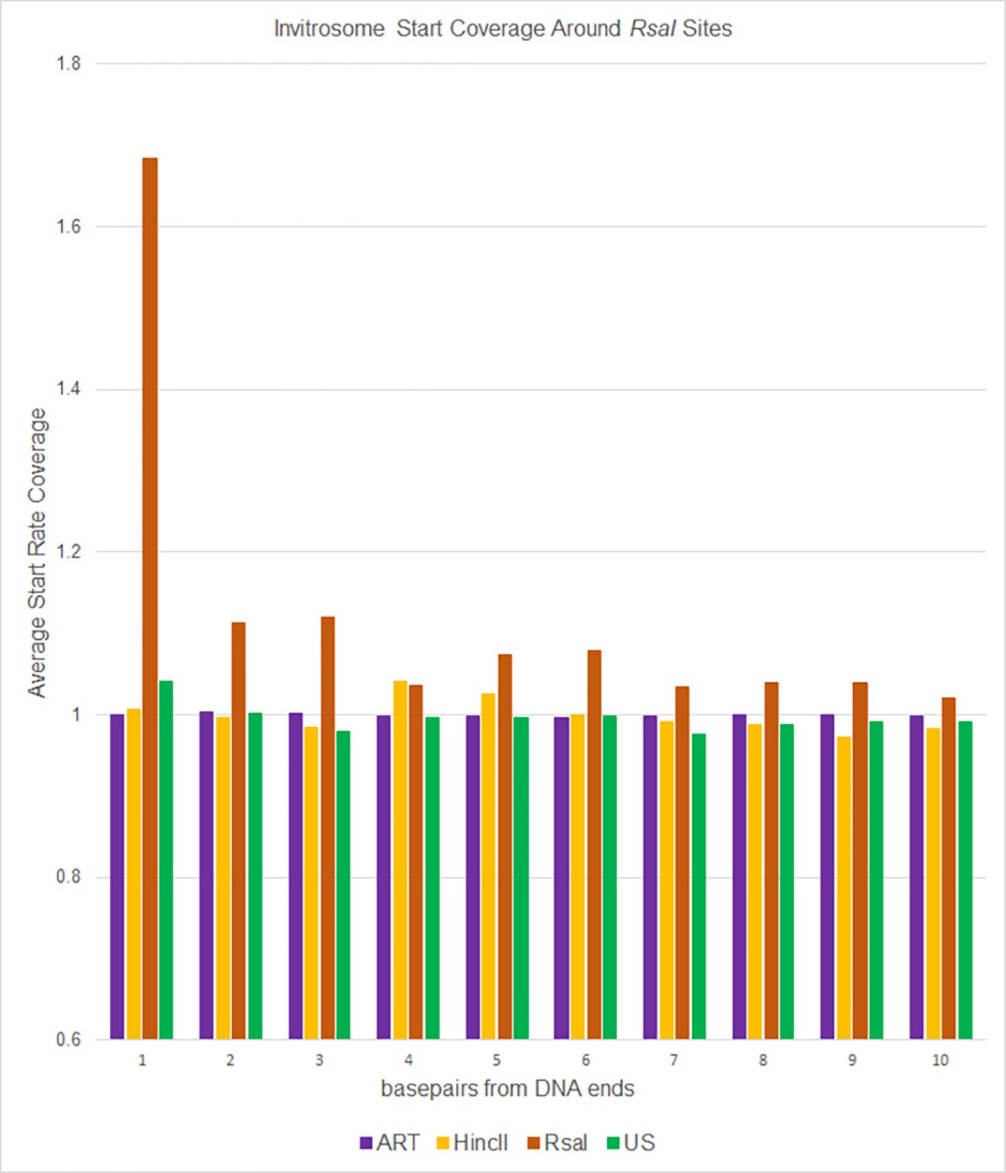
RsaI occupancy of positions. Average occupancy (or coverage) of invitrosome starts around *RsaI* restriction sites in the genome for each of the four invitrosome data sets (ART, HincII, RsaI and US). Positions 1 through 10 on the x-axis are relative to *RsaI* cut sites across the genome. A value of 1 indicates occupancy at an average rate across the genome. Higher than 1 indicates above average occupancy.

**Fig 8:**
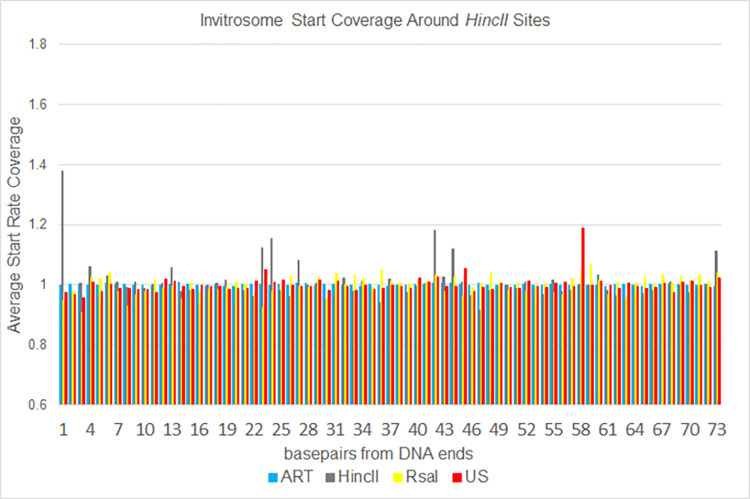
Expanded Fig 6. The same data as shown in Fig 6 expanded out to 73 bases from genomic *HincII* cut sites.

**Fig 9:**
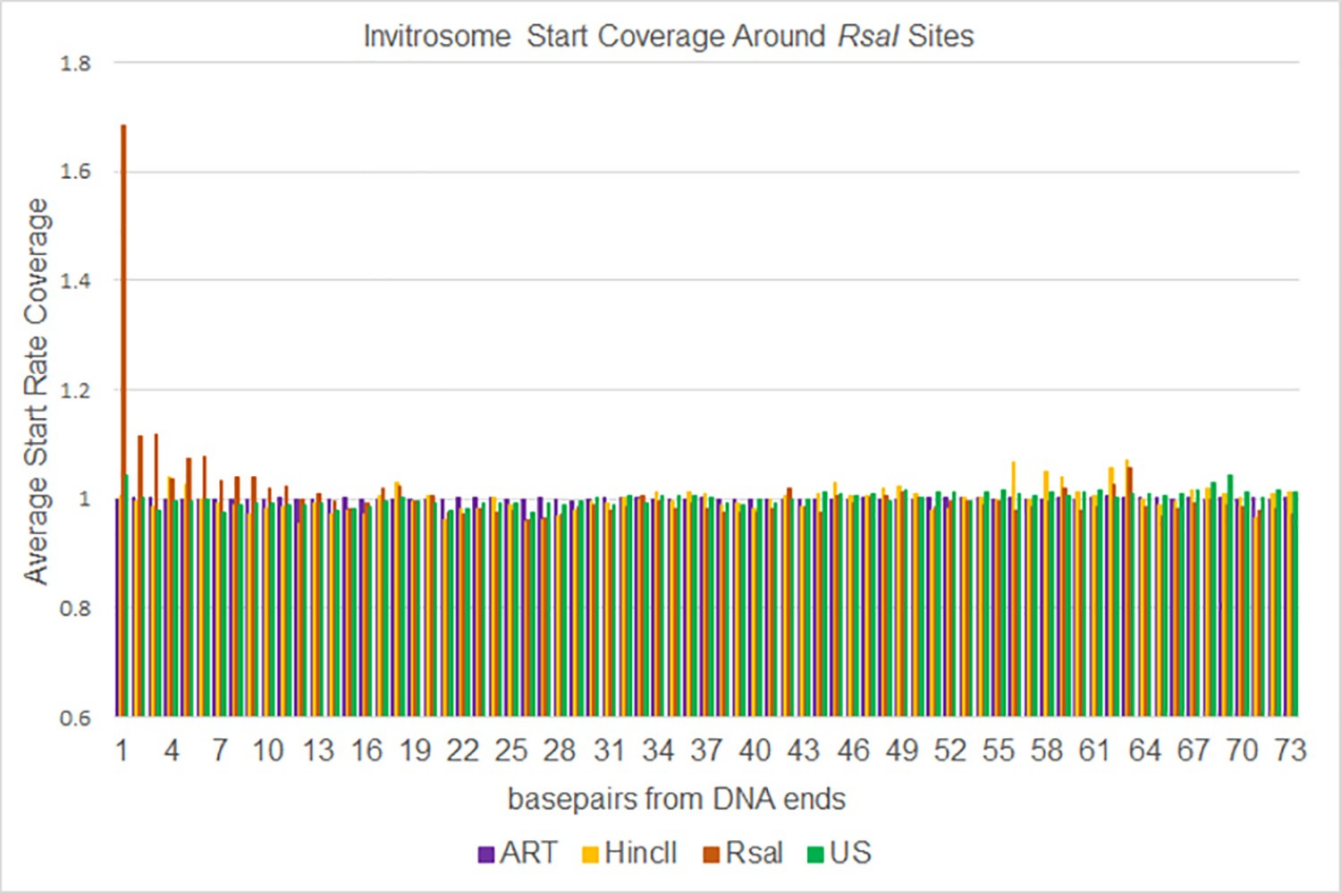
Expanded Fig 7. The same data as shown in Fig 7 expanded out to 73 bases from genomic *RsaI* cut sites.

To analyze the data a different way, we calculated invitrosome start coverage values for the same libraries, starting at position 1 out to position 73 using bamCoverage from the deepTools2 [63] suite. We calculated a mean coverage, and then took the coverage of each position divided by the mean coverage and graphed them out to 10 bp and out to 73 bp. We saw in the *RsaI* and *HincII* datasets an unusually high occupancy value at the first position around their own respective cutsites, that quickly approached a value of 1 within a few positions of moving in from the end (Figs 6 and 7). The values fluctuated around 1 for the remaining length of the 73 bp (Figs 8 and 9). It is important to note that the other libraries, such as ART and US, had values around 1 beginning at the first position regardless of which cut site was used for the analysis, demonstrating that end bias is indeed due to the physical ends of DNA fragments and not due to positioning signals in the *C*. *elegans* genome around those cutsites. Also, analysis of the *RsaI* dataset using *HincII* cutsites and the *HincII* dataset using *RsaI* cutsites both resulted in values around 1 at position 1, further confirming this conclusion. From both sets of analyses, we found that end bias accounts for anywhere from, at the low end, a 40% increase, to at the high end, up to a 300% increase in invitrosome formation at the end of DNA fragments.

We were curious as to what might explain this end bias phenomenon. Based on the simulations of Sakaue et al. [9] nucleosomes would form on the end due to steric hindrance of two DNA helices at both DNA entry/exit sites. Forming exactly on the end of DNA would only produce one DNA helix at the DNA entry/exit sites, thereby reducing steric hindrance. They followed their simulations with their own invitrosome experiments and visualized the invitrosomes on the ends of their DNA at a rate of near 100 percent. However, we think their hypothesis of the cause of end bias unlikely. Firstly, the researchers never mention what sort of DNA they use for their reconstitutions. It is unknown whether it was PCR amplified DNAs, fragmented genomic DNA, or linearized plasmid. Research has demonstrated that the specific DNA sequence is extremely important, especially *in vitro*. Their results could have been substantially influenced by their choice of DNA. Secondly, when assembling invitrosomes, the H3/H4 tetramer binds the DNA first, followed by the first H2A/H2B dimer, followed by the second dimer [64]. The tetramer would have to “sense” in some way when interacting with the DNA that the fully formed invitrosome would not have the steric hindrance of two helices. Finally, their simulations used tailless histones, which do not represent the majority of invitrosomes. While steric hindrance may be a problem for tailless nucleosomes, histone tails interact with linker DNA and “pull” it in to form a tighter nucleosome [65], potentially eliminating or reducing such hindrance.

There are a few other alternatives. First, there could be some slight invitrosome shifting laterally along the DNA once it is formed. Potentially that could eliminate the steric hindrance of two DNA helices. Secondly, there might be some torsional strain forced onto the DNA as the invitrosome forms that is quickly relieved when the invitrosome happens to be near the end of the DNA, thus lowering the free energy of formation. Analysis of the crystal structure of nucleosomes has revealed unique perturbations in the DNA’s path around the nucleosome not seen in other DNA binding proteins [66]. It is plausible that perturbations, such as excessive DNA curvature and alternating DNA forms, could induce torsional strain. Since there are not areas of reduced invitrosome occupancy across the DNA that we could detect in this study, we think that if torsional strain is the cause, the torsional strain is minimal and that there is only slightly more favorable free energy at the end of the DNA fragment that quickly reaches a baseline free energy level moving towards the DNA fragment center. Lastly, end bias could be a result of the experimental procedure. Invitrosome formation requires high salt levels at the beginning to balance out the negative charge of the DNA and positive charge of the protein. Ions are slowly reduced, and the DNA and histone proteins come together. Perhaps the DNA ends allow tetramer or dimer binding at a slightly higher ion concentration than the center of the DNA does.

### Comparative analysis allows the recovery of suspect invitrosome reads

Having demonstrated that invitrosomes form on DNA fragment ends up to four times as frequently as they would normally form due to simply DNA sequence favorability, we wanted to be able to distinguish between invitrosomes formed due to end bias versus invitrosomes formed on the ends of DNA fragments due to DNA sequence preference. To this end we devised a method to accomplish this based on the use of the two defined-end invitrosome libraries used above (*RsaI* and *HincII*).

Currently using conventional approaches, two classes of DNA loci are typically excluded from invitrosome analyses or have invitrosomes discarded in order to eliminate potential end bias. When DNA fragment ends are defined, 1) any invitrosome found to map within a defined number of nucleotides from a DNA fragment end is classified as suspect of end bias and is discarded. 2) DNA fragments digested to sizes too small for reconstitution (<147 bp) are lost from invitrosome analyses (Fig 1).

We hypothesized that both of these classes of excluded loci can potentially be recovered and analyzed by performing nucleosome reconstitutions on two DNA samples digested by two different restriction endonucleases. Each individually-digested DNA sample is used for separate nucleosome reconstitutions and then invitrosome positions from the two libraries are identified by mapping sequenced mononucleosome core DNAs back to the original source of DNA. For each individual library, invitrosomes that may suffer from end bias can be identified by defining a specific number of bases from cutsites (DNA fragment ends) as “too close” to the end of the DNA fragment (the suspect range). Invitrosomes that map and start within suspect-range regions are considered theoretically subject to end bias and so are defined as “suspect” invitrosomes. Invitrosomes that do not fall within the suspect-range regions are assumed to not be affected by end bias and are classified as “passed” nucleosomes.

The restriction sites of the two restriction endonucleases used will usually not be near one another on the DNA. Therefore, invitrosomes from one library that are defined as suspect and normally would be discarded (due to proximity to a DNA fragment end) can be recovered if the same locus is found to be occupied by a “passed” invitrosome in the second library. This is demonstrated in Fig 1 with the example invitrosomes in position 3 and position 4. In contrast, in Fig 1, invitrosomes in position 2 remain in doubt as this position is near a DNA fragment end in both experiments and both invitrosomes are “suspect”.

Additionally, the positions where DNA fragments were generated that were too small to participate in reconstitution can be recovered; as the likelihood of this happening with both endonuclease digestions is small; a position lost in one experiment can be recovered if in the second experiment the fragment is of sufficient size to form a “passed” invitrosome (e.g., Fig 1 position 1).

We applied this recovery method to the invitrosome libraries generated using the *C*. *elegans* genome described in Locke et al. which were reconstituted on DNA that was digested with the *RsaI* or the *HincII* restriction enzymes. Our recovery approach is composed of three steps. 1) a suspect range is generated based on a user-defined variable, 2) invitrosomes are mapped and declared either passed or suspect, and 3) suspect invitrosomes are recovered by comparison to the alternate experiment’s set of passed invitrosomes. In applying the first step, generation of suspect-range regions is dependent on knowing precise fragment ends produced by restriction enzyme digestion. Because two different restriction endonucleases are used, the loci that fall into the suspect-range regions will be different for the two experiments and will depend on the restriction endonuclease used to prepare the template DNA for reconstitution. We used the fragment-end list generated by Locke et al. to define the beginning and end of DNA fragments based on the presence of either a *RsaI* or a *HincII* cut site. This list contains the start, end and fragment size of all hypothetical fragments generated across all chromosomes by digestion with these enzymes [8]. In the Locke analysis the suspect range was defined as 200 bp from a DNA fragment start and 200 bp from the fragment end, a total range of 400 bp per DNA fragment. We used the same 200-bp suspect range to be consistent with the results of the Locke analysis. To generate each suspect-range region, the genomic position of each DNA-fragment start or DNA-fragment end (excluding the palindromic restriction enzyme cut site) had the suspect range-defined number of base pairs added to or subtracted from it respectively, producing suspect-range-defined starts or ends. This resulted in unique sets of suspect-range regions across the genome for each restriction enzyme.

We applied the second step of our approach by mapping all the invitrosome sequence reads from both experiments to the WS190 version of the *C*. *elegans* genome. After mapping the sequence reads, each read was extended out to 147 bp to represent the entire footprint of the invitrosome from which it was derived, and the direct center, or dyad position, was recorded to produce sets of both *HincII*-invitrosome dyads and *RsaI*-invitrosome dyads. During analysis, 73 bp was added and subtracted from the dyad to produce start and end positions for both sets. Start and end positions were then compared to their respective suspect-range regions. Depending on where each invitrosome end fell relative to the suspect-range regions (within the suspect range or outside of the suspect range), it was defined as either “suspect” or “passed” respectively. Any invitrosome with a start that fell into suspect-range start region or any invitrosome with an end that fell into a suspect-range end region was defined as “suspect.” Passed invitrosomes were separated from suspect invitrosomes and kept as unbiased data for each experiment. Putative underdigested DNA fragments which had invitrosome reads that tiled over cut sites were also considered passed but kept separate for statistical purposes (InnerCutSite). For each experiment the suspect-range size was kept the same between the *RsaI* and the *HincII* datasets. This resulted in six invitrosome sets from the two experiments: passed-*RsaI* invitrosomes, suspect-*RsaI* invitrosomes, InnerCutSite-*RsaI* invitrosomes, passed-*HincII* invitrosomes, suspect-*HincII* invitrosomes, and InnerCutSite-*HincII* invitrosomes.

**Fig 10:**
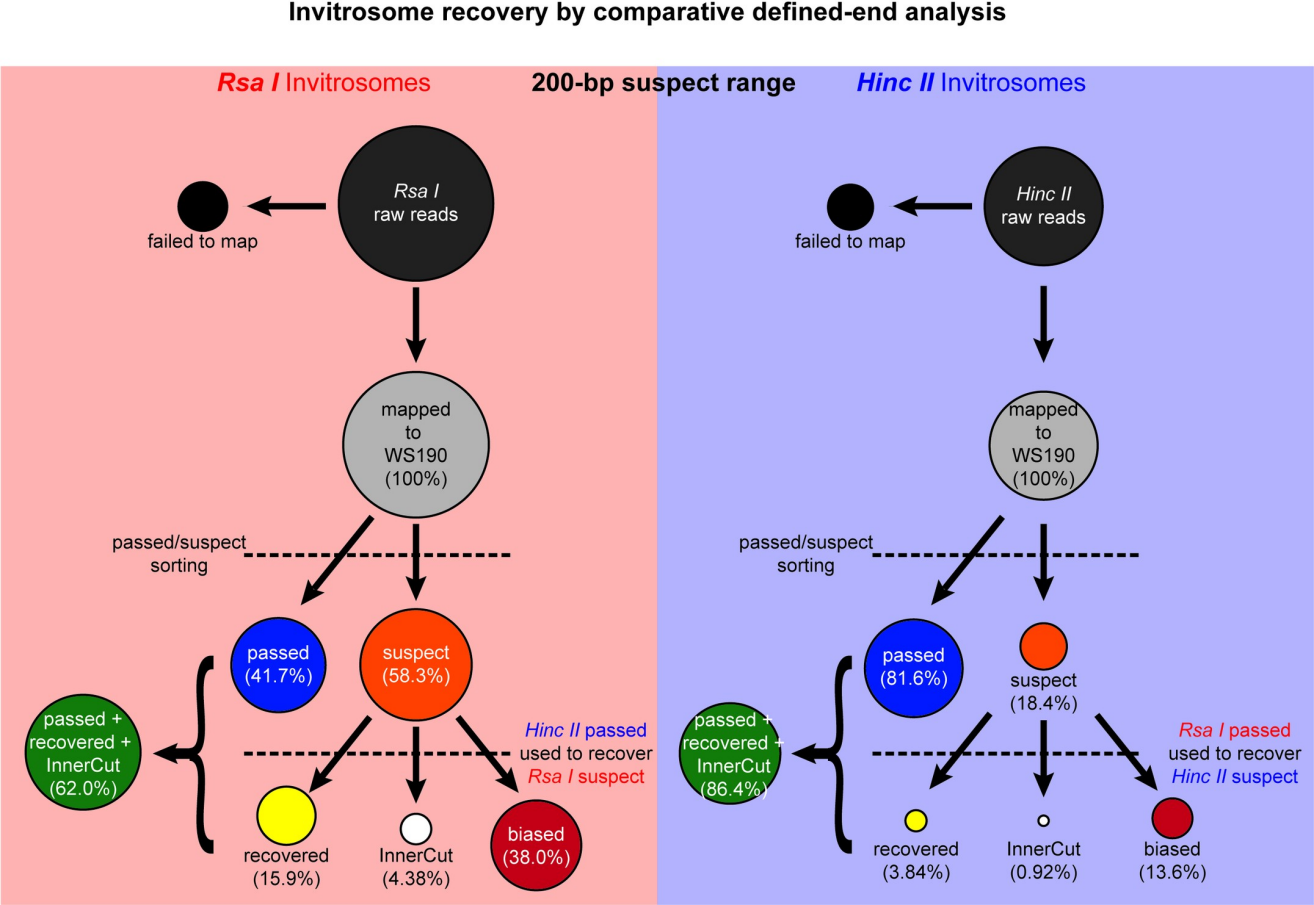
Reads suspected, recovered, and tossed at 200 bp. Overview of the recovery method to “rescue” invitrosomes suspected of end bias. Depiction of the percentages of reads, and by extension invitrosomes, during the classification and re-classification method. Invitrosomes too close to DNA fragment ends (in this case within 200 base pairs) were deemed suspect to formation on fragment ends due to end bias. Invitrosomes outside of 200 base pairs from DNA ends were deemed as passed; while invitrosomes containing a cutsite for the respective restriction enzyme within the nucleosomal DNA would normally be discarded are recovered. Using the alternate library’s passed invitrosomes, suspect invitrosomes were re-classified as recovered if a passed invitrosome from the other library could be found at the same position as the suspect invitrosome. The comparative analysis increases the sequencing data available for downstream analysis.

The final step was to recover suspect invitrosomes from one experiment and reclassify them as free of end bias through comparison with passed invitrosome reads from the alternate experiment. Suspect-*RsaI* invitrosomes were compared to passed-*HincII* invitrosomes and InnerCutSite-*HincII* invitrosomes, while suspect-*HincII* invitrosomes were compared to passed-*RsaI* invitrosomes and InnerCutSite-*RsaI* invitrosomes. Suspect invitrosomes that start at the same position as passed invitrosomes from the alternative fragment set were now reclassified as “recovered” invitrosomes. Those that did not receive this new classification are considered to be potentially affected by end bias and were reclassified as “biased” invitrosomes. The final result is a set of recovered and biased invitrosomes for each library. The results generated by the entire workflow were eight unique output files: passed-*RsaI* invitrosomes, InnerCutSite-*RsaI* invitrosomes, recovered-*RsaI* invitrosomes, biased-*RsaI* invitrosomes, passed-*HincII* invitrosomes, InnerCutSite-*HincII* invitrosomes, recovered-*HincII* invitrosomes, and biased-*HincII* invitrosomes. The complete workflow is shown in Fig 10.

### Recovery of *RsaI* and *HincII* invitrosomes

The mapped *RsaI* dataset contained a total of 8,463,490 invitrosomes. Using our 200-bp suspect range; 4,933,904 or 58.3% of the mapped *RsaI* invitrosomes were declared suspect (Table 1 and Fig 10). Without our recovery method these suspect invitrosomes would be lost to further analysis.

**Table 1:** Reads before and after recovery.

	*HincII*	*RsaI*	* *	* *	* *	* *
Total Reads	5,537,994	9,489,038				
Mapped Reads	4,653,842	8,463,490				
	84%*	89.2%*				
	200 bp		11 bp		1 bp	
	*HincII*	*RsaI*	*HincII*	*RsaI*	*HincII*	*RsaI*
Passed	3,799,051	3,529,586	4,531,053	7,227,702	4,582,097	7,725,507
	81.6%	41.7%	97.4%	85.4%	98.5%	91.3%
All Suspect	854,791	4,933,904	122,789	1,235,770	71,745	737,983
	18.4%	58.3%	2.6%	14.6%	1.5%	8.7%
Inner Cut Site	43,111	370,753	43,111	370,753	43,111	370,753
	0.9%	4.4%	0.9%	4.4%	0.9%	4.4%
Recovered	178,865	1,348,245	38,484	291,526	14,757	120,562
	3.8%	15.9%	0.8%	3.4%	0.3%	1.4%
Total Recovered	221,976	1,718,998	81,595	662,279	57,868	491,315
	4.8%	20.3%	1.8%	7.8%	1.2%	5.8%
Remaining suspect	632,815	3,214,906	41,194	573,491	13,877	246,668
	13.6%	38.0%	0.9%	6.8%	0.3%	2.9%
Post Recovery	4,021,027	5,248,584	4,612,648	7,889,981	4,639,965	8,216,822
	86.4%	62.0%	99.1%	93.2%	99.7%	97.1%

Absolute counts and percentages of reads before and after the recovery program.

* Represent percentages taken from Total Reads. All other percentages are taken from Mapped Reads.

In order to recover suspect-*RsaI* invitrosomes we compared these invitrosomes to the passed-*HincII* invitrosomes that were analyzed at the *HincII* 200-bp suspect range. As described above, any suspect-*RsaI* invitrosome that shared the same position with a passed-*HincII* invitrosome was assumed to be an invitrosome that formed at that particular locus due to preferable DNA sequence rather than end-position bias and was declared recovered. This comparison resulted in 1,348,245 (15.9%) of the suspect-*RsaI* invitrosomes being reclassified as recovered through comparison, and 370,753 (4.4%) invitrosomes being reclassified as formed on underdigested DNA and recovered (InnerCutSite). Thus using our recovery method we recovered 20.3% of the suspect-*RsaI* invitrosomes resulting in a total of 5,248,584 passed- or recovered-*RsaI* invitrosomes, or 62.0% of the original mapped invitrosome set. This left 3,214,906 suspect invitrosomes that were reclassified as biased and unusable, which is 38.0% of the original *RsaI* mapped invitrosome set after processing, instead of the 58.3% that would be unusable without our recovery procedure (Fig 10).

The same analysis was performed on the 4,653,842 mapped *HincII* invitrosomes, with recovery analysis being performed with the passed-*RsaI* invitrosomes that were analyzed at the *RsaI* 200-bp suspect range. At the suspect range of 200 bp; 854,791 (18.4%) of the *HincII* invitrosomes were declared suspect (Fig 10). Using the passed-*RsaI* invitrosomes, 178,865 (3.8%) suspect-*HincII* invitrosomes were recovered through comparison and 43,111 (0.9%) suspect-*HincII* invitrosomes were recovered through underdigested recovery. The remaining 632,815 (13.6%) suspect-*HincII* invitrosomes were labeled as biased. Thus using our recovery method we recouped 20.9% of the suspect-*HincII* invitrosomes through comparison and 5.0% of the suspect-*HincII* invitrosomes through underdigested recovery, for a total of 26.0% of the biased reads recovered. A total of 4,021,027 (86.4%) passed- or recovered-*HincII* invitrosomes, of the original invitrosome set (Fig 10) were usable after our recovery approach. The remaining 632,815 biased invitrosomes represent 13.6% of the original mapped *HincII* invitrosome set that was still unusable (Fig 10). Despite the more modest size of this recovery, it still represents a substantial improvement over the 18.4% that would be unusable without our recovery procedure.

### Varying the suspect range length

We wanted to test the effect of varying lengths of suspect ranges on the number of invitrosomes declared suspect and recovered by our approach. To this end, we applied two more suspect ranges: 1 bp and 11 bp (one helical turn of DNA). We compared the results of applying these additional two suspect ranges to the results from our 200-bp suspect range. As expected, with decreased suspect range size we see a decrease in the number of suspect invitrosomes. Specifically, we see the number of suspect invitrosomes decrease in relation to the length of the suspect range, with the lowest suspect range of a single base pair resulting in a low of only 737,983 (8.7%) of the *RsaI* and 71,745 (1.5%) of the *HincII* invitrosomes being declared suspect (S1 and S2 Figs and Table 1). It is interesting to note that for *RsaI* invitrosomes, at the larger suspect range of 200 bp, the number of suspect invitrosomes is actually greater than the number of passed invitrosomes. This is not the case for the *HincII* invitrosomes. This is due to the frequency of the restriction sites occurring in the genome. On average, *RsaI* sites occur every 490 base pairs and *HincII* sites would occur every 2109 base pairs [8]. Using a 200-bp suspect range would render an alarming 81.6% and 19.0% of the genome suspect for *RsaI* and *HincII*, respectively.

The 11-bp suspect range is of particular interest as it represents one full turn of the DNA helix. If invitrosomes were to be affected by end bias, but still try and retain a preferential rotational setting, it might be predicted that they would form between 1–11 bp from the end of the DNA fragment as this would cover all potential rotational settings. Interestingly, previous studies have demonstrated that virtually all end-effect nucleosome positioning results in invitrosomes within about ±10 bp of the fragment end [10]. At the 11-bp suspect range 1,235,770 (14.6%) of mapped *RsaI* invitrosomes are suspect and 122,789 (2.6%) of mapped *HincII* invitrosomes are suspect (S1 Fig). At this same level, 662,279 (53.6%) of the suspect-*RsaI* are recovered, with 291,526 saved through comparison and 370,753 saved through underdigested comparison (S1 Fig). 81,595 (66.5%) of suspect-*HincII* invitrosomes are recovered, with 38,484 through comparison and 43,111 through underdigested comparison (S1 Fig and Table 1).

Having applied our approach, we find that a substantial number of suspect invitrosomes can be recovered within the *RsaI* invitrosome set no matter what size the suspect range is. Within the maximum 200-bp suspect range we find that our approach is able to recover 34.8% or 1,718,998 of the suspect-*RsaI* invitrosomes. However, with the smaller 11-bp suspect range, we are able to recover 53.6% or 662,279 of the suspect-*RsaI* invitrosomes.

### End bias does not significantly skew 4-mer results

Often one of the major goals of invitrosome experiments is to identify and analyze the DNA sequence preferences that guide nucleosome positioning *in vitro* and compare that with such DNA signals *in vivo*. Having demonstrated substantial end bias in invitrosome experiments, we wanted to see what effect end bias has on the DNA sequences that are seen in invitrosomes. With our recovery method, we were able to compare invitrosome DNA sequences (specifically k-mer usage) from total data sets, non-suspect data sets and post recovery data sets.

**Fig 11:**
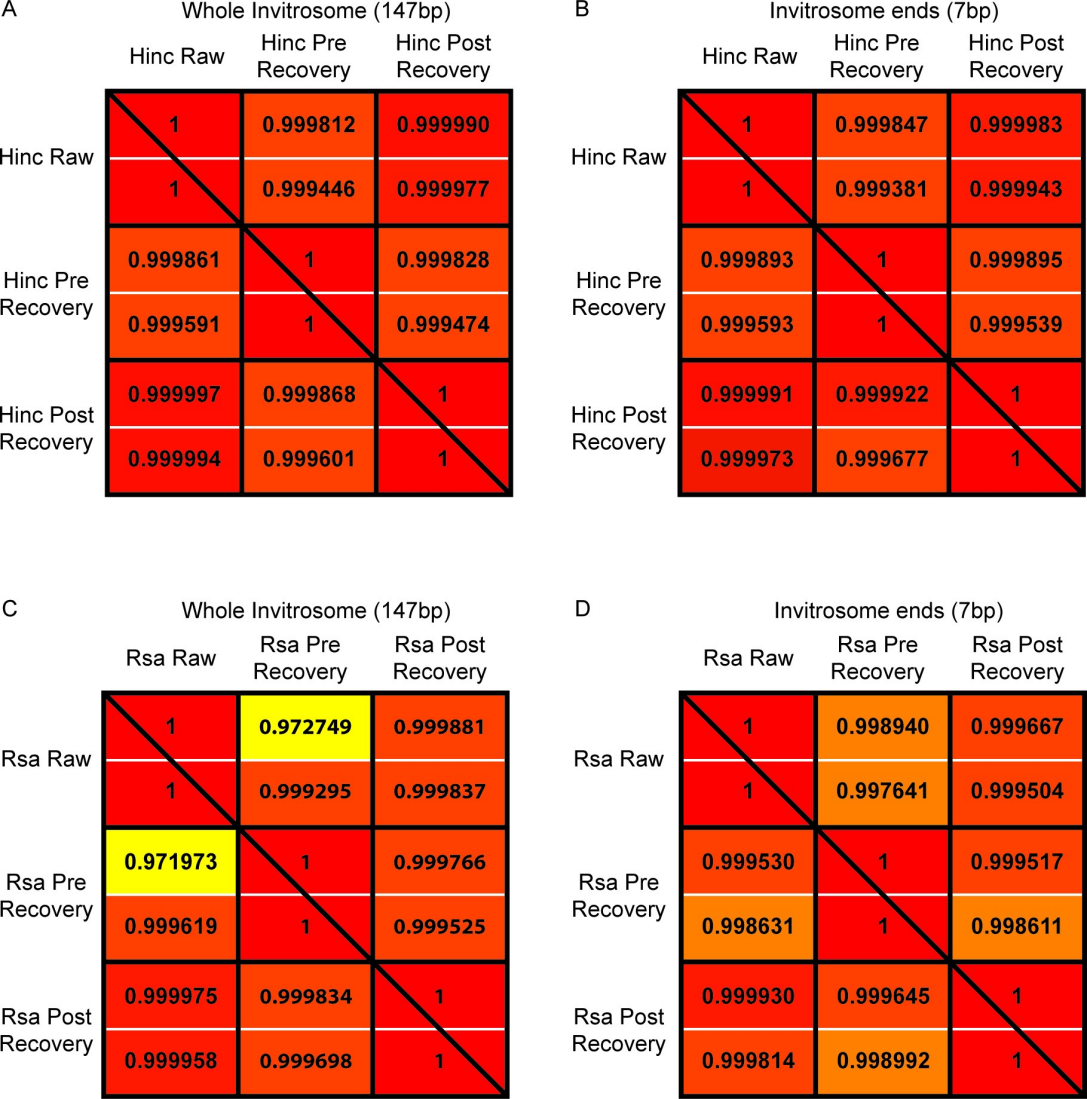
Correlations within sub-libraries. Correlation between k-mers at each step of recovery. Correlation values for k-mer usage from the *HincII* data are shown in panels A) and B). Correlation values for k-mer usage from the *RsaI* data are shown in panels C) and D). In all panels, within each black box, the Pearson correlation value is listed above, and the Spearman correlation value is listed below the white line. Correlation values in the bottom-left-triangle half of the entire panel are between sub-libraries with a 1-bp suspect range, and values in the top-right-triangle half are between sub-libraries with a 11-bp suspect range. Panels A) and C) are k-mer usage correlation values calculated across the entire 147-bp invitrosome DNA, while panels B) and D) are k-mer correlation values looking at k-mer usage only at the ends of invitrosome DNA.

With our libraries mapped to the reference genome, we extrapolated out the DNA sequences of the invitrosomes and calculated the frequency of 4-mers for each library in a position dependent manner across the invitrosome DNA cores. We then took the rate of the various 4-mer usages and measured the differences between the libraries using two different correlation methods. Correlation values were calculated between the enzyme libraries (*RsaI* and *HincII*) and the non-enzyme libraries (ART and US), utilizing both the Pearson and Spearman correlation methods. We first asked if 4-mer frequency was different across the entire 147 base pairs of nucleosomal DNA, comparing the data from the raw libraries to that of the libraries at each recovery step. We did this for both suspect regions of 1 bp or 11 bp from the end (Table 2). Our previous analyses demonstrated that end bias is only seen within the first few bases of DNA fragment ends, thus we did not do similar correlations with the 200-bp suspect region data. Regardless of which correlation method was used, correlation values between each enzyme library compared to the non-enzyme libraries at each step of the recovery process (i.e., each sub-library) differed by a trivial amount. Wondering if differences were being hidden based on the amount of data in the correlation calculations due to using all 147 base pairs of the invitrosome cores, we narrowed our analysis to the 7 positions on either end of the invitrosome DNA (where we would expect to find end biased invitrosomes), functionally reducing the amount of data down an order of magnitude. Again, regardless of which correlation method was used, the differences were trivial (Table 3). Additionally, we calculated the correlations between the individual sub-libraries within the *RsaI* or *HincII* libraries at each step of the recovery process. As expected, both correlation methods showed even less difference between the various sub-libraries with all correlations being above 0.97 (Fig 11). Considering there is little relevant change in the correlation values between the various classifications of invitrosomes, regardless of the correlation method used, we conclude that any changes in the 4-mer composition from invitrosome end bias is negligible.

**Table 2:** Correlations of known ends to unknown end libraries.

1 bp Suspect Ends		Pearson			Spearman	
		ART	US		ART	US
	Hinc Raw Reads	0.8647	0.9386	Hinc Raw Reads	0.7861	0.8900
	Hinc Passed	0.8640	0.9381	Hinc Passed	0.7845	0.8891
	Hinc Post Recovery	0.8647	0.9385	Hinc Post Recovery	0.7859	0.8898
	Rsa Raw Reads	0.9017	0.9672	Rsa Raw Reads	0.8902	0.9527
	Rsa Passed	0.9000	0.9657	Rsa Passed	0.8878	0.9506
	Rsa Post Recovery	0.9013	0.9668	Rsa Post Recovery	0.8895	0.9522
11 bp Suspect Ends		Pearson			Spearman	
		ART	US		ART	US
	Hinc Raw Reads	0.8647	0.9386	Hinc Raw Reads	0.7861	0.8900
	Hinc Passed	0.8645	0.9382	Hinc Passed	0.7841	0.8887
	Hinc Post Recovery	0.8645	0.9382	Hinc Post Recovery	0.7852	0.8893
	Rsa Raw Reads	0.9017	0.9672	Rsa Raw Reads	0.8902	0.9527
	Rsa Passed	0.9012	0.9664	Rsa Passed	0.8882	0.9512
	Rsa Post Recovery	0.9001	0.9666	Rsa Post Recovery	0.8872	0.9516

Correlation values for 4-mer frequencies across all positions of nucleosomal DNA for two different suspect region lengths. There are very small differences between the Raw aligned, Non-suspect or passed, and Post Recovery reads when compared to the ART dataset and US dataset.

**Table 3:** Correlations of known ends to unknown end libraries.

1 bp Suspect Ends		Pearson			Spearman	
		ART	US		ART	US
	Hinc Raw Reads	0.8450	0.8651	Hinc Raw Reads	0.6787	0.7550
	Hinc Passed	0.8449	0.8647	Hinc Passed	0.6778	0.7541
	Hinc Post Recovery	0.8449	0.8648	Hinc Post Recovery	0.6785	0.7544
	Rsa Raw Reads	0.9106	0.8981	Rsa Raw Reads	0.8227	0.8168
	Rsa Passed	0.9093	0.8965	Rsa Passed	0.8174	0.8131
	Rsa Post Recovery	0.9097	0.8973	Rsa Post Recovery	0.8214	0.8155
11 bp Suspect Ends		Pearson			Spearman	
		ART	US		ART	US
	Hinc Raw Reads	0.8450	0.8651	Hinc Raw Reads	0.6613	0.7395
	Hinc Passed	0.8443	0.8643	Hinc Passed	0.6592	0.7380
	Hinc Post Recovery	0.8444	0.8646	Hinc Post Recovery	0.6602	0.7386
	Rsa Raw Reads	0.9106	0.8981	Rsa Raw Reads	0.8118	0.8034
	Rsa Passed	0.9116	0.9000	Rsa Passed	0.8097	0.8069
	Rsa Post Recovery	0.9100	0.8998	Rsa Post Recovery	0.8095	0.8066

Correlation values of 4-mer frequencies for the 7 positions from both ends of nucleosomal DNA for two different suspect regions lengths. There are very small differences between the Raw aligned, Non-suspect or passed, and Post Recovery reads when compared to the ART dataset and US dataset.

### End bias does not affect further down-stream analysis

**Fig 12:**
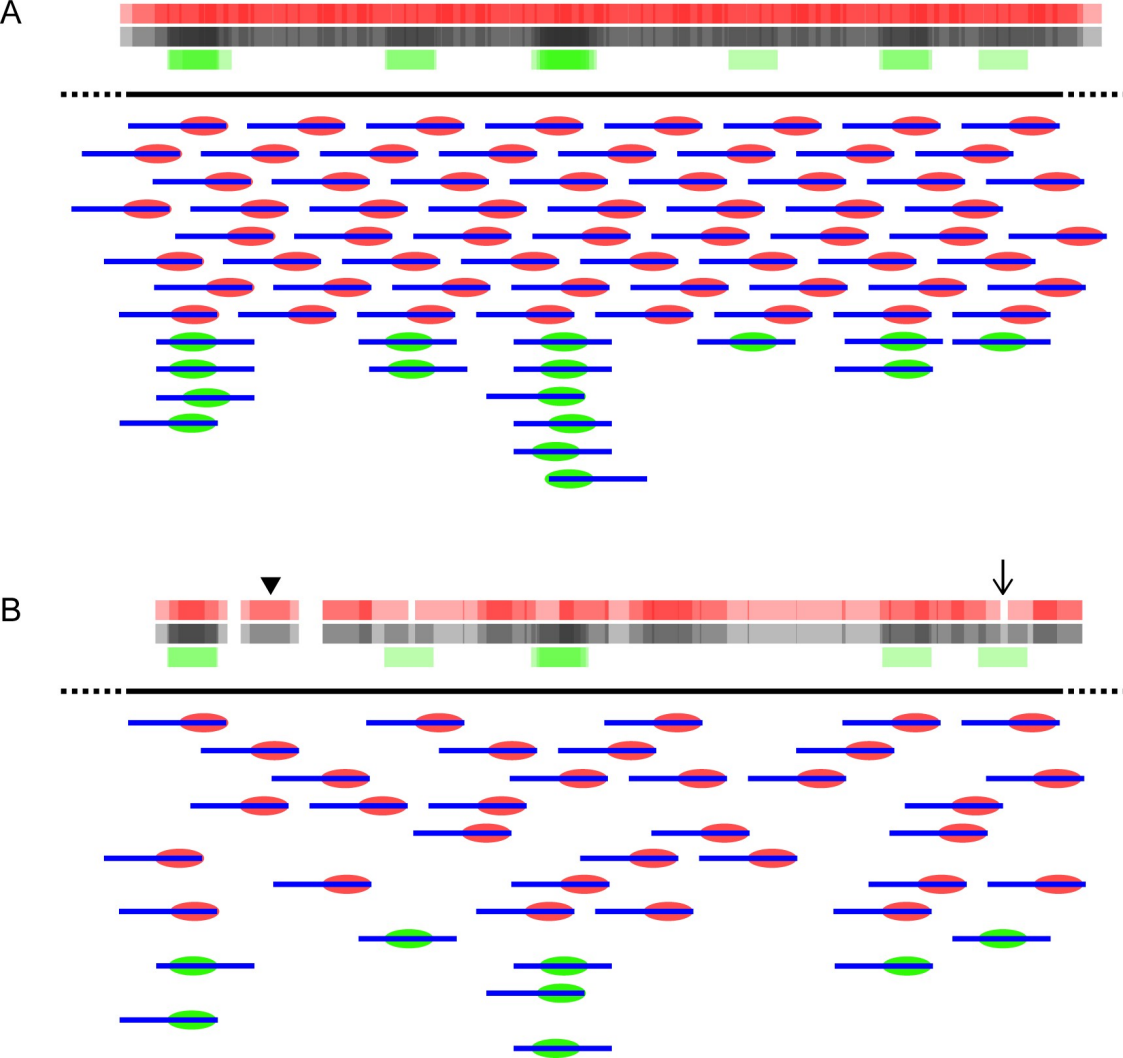
Role of sequencing depth and end bias. A) Regardless of proximal-end bias taking place, with adequate sequencing depth, the number of *in vitro* reconstituted nucleosomes (invitrosomes) formed and their proximity to each other prevent underlying sequence bias from obfuscating invitrosomes positioned by DNA sequence. B) With inadequate sequencing depth, the sparse invitrosome coverage in conjunction with proximal-end bias would produce erroneous invitrosome positioning data (black arrowhead) while missing real invitrosome positioning DNA sequences (arrow). In both panels red ovals represent invitrosomes affected by end bias and green ovals represent invitrosomes formed due to DNA sequence preference. Blue lines represent the DNA fragments upon which the invitrosomes have formed. The red bars, green bars and black bars at the top of each panel represent invitrosome occupancy density from end-biased, DNA-sequence positioned, and the combined invitrosome data respectively.

Three things are important to note in addition to the conclusions from our analyses. First, that in the Locke et al. analysis, they failed to see a significant difference in invitrosome occupancy on gene elements, such as exons and introns, when they discarded potentially biased data. This adds further to the claim that while end bias exists, it does not alter data interpretation. Second, sequencing depth could also play a role in any observed bias (Fig 12). With adequate sequencing depth, any bias could be “drowned out”. With insufficient depth, the bias could become substantial. One possibility is that bias was not observed due to the sequencing depth. Within a single genome where k-mer usage is present throughout the genome, the required depth of coverage to compensate for end bias need not be across the entire genome but rather simply represented in the random reads sequenced. However, with today’s high throughput sequencing, many more reads are typically obtained compared to the number used in this study; we therefore assume that few if any researchers in the future will have a problem with end bias skewing data interpretation. Lastly, invitrosome methods are not as universal as some may think. A lot of variables can differ between labs and even experiments, such as the speed at which the ion concentrations are reduced, linear vs stepwise ion reduction, ratio of DNA to protein, and so on. Differences in these variables between labs could change end bias outcomes. For example, higher concentrations of histone octamer relative to DNA would skew invitrosome formation towards the center of DNA fragments. For such a scenario, in theory the DNA ends would all be occupied with invitrosomes, forcing formation much further from the DNA ends. For the sonicated DNA invitrosome reconstitution used in this study, we followed the exact same procedure and ratios that Locke et al. used in their experiments, which generated the *RsaI* and *HincII* datasets. Perhaps in our analysis the consistency in methodology used does not skew the underlying data, but other methods might.

## Supporting information

S1 Fig Reads suspected, recovered, and tossed at 11 bp. Visual depiction of the percentages of reads, and by extension invitrosomes, during the classification and re-classification method. Invitrosomes too close to DNA fragment ends (in this case within 11 base pairs) were deemed suspect to formation on fragment ends due to end bias. Invitrosomes outside of 11 base pairs from DNA ends were deemed as passed, while invitrosomes containing a cutsite for the respective restriction enzyme within the nucleosomal DNA would normally be discarded are recovered. Using the alternate library’s passed invitrosomes, suspect invitrosomes were re-classified as recovered if a passed invitrosome from the other library could be found at the same position as the suspect invitrosome. The comparative analysis increases the sequencing data available for downstream analysis.
(TIF)

S2 Fig Reads suspected, recovered, and tossed at 1 bp. Visual depiction of the percentages of reads, and by extension invitrosomes, during the classification and re-classification method. Invitrosomes too close to DNA fragment ends (in this case within 1 base pair) were deemed suspect to formation on fragment ends due to end bias. Invitrosomes outside of 1 base pair from DNA ends were deemed as passed, while invitrosomes containing a cutsite for the respective restriction enzyme within the nucleosomal DNA would normally be discarded are recovered. Using the alternate library’s passed invitrosomes, suspect invitrosomes were re-classified as recovered if a passed invitrosome from the other library could be found at the same position as the suspect invitrosome. The comparative analysis increases the sequencing data available for downstream analysis.
(TIF)

S1 Table Program parameters. Table of publicly available programs used in the analysis. All parameters for programs were set to default except as noted.
(TIF)
