# Supplementary figures and images for: Proximal-end bias from in-vitro reconstituted nucleosomes and the result on downstream data analysis
Source: PLoS One. 2021 Oct 21;16(10):e0258737. doi: 10.1371/journal.pone.0258737 (PMC8530345; doi:10.1371/journal.pone.0258737)

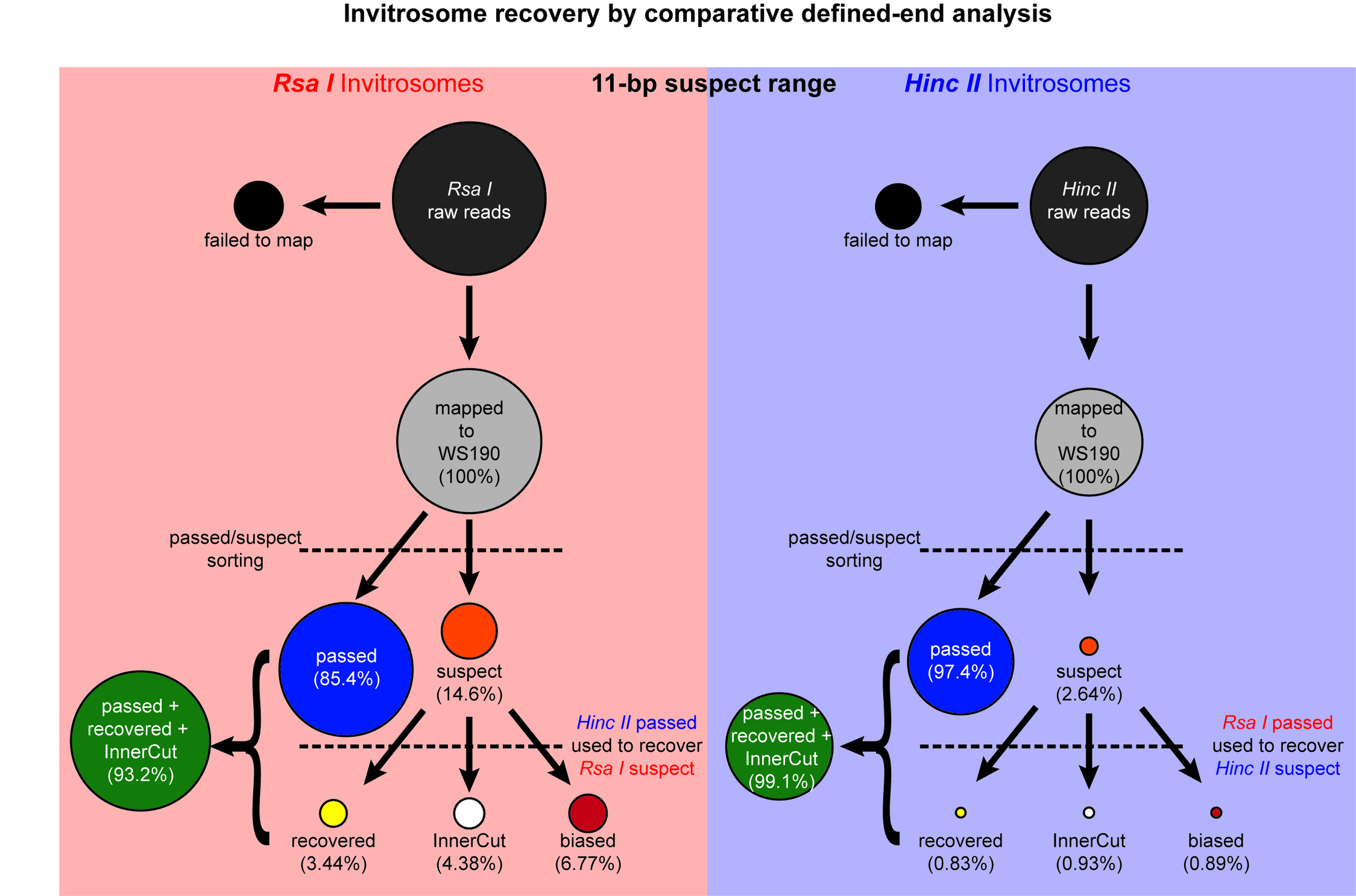

Supplement: S1 Fig — Visual depiction of the percentages of reads, and by extension invitrosomes, during the classification and re-classification method. Invitrosomes too close to DNA fragment ends (in this case within 11 base pairs) were deemed suspect to formation on fragment ends due to end bias. Invitrosomes outside of 11 base pairs from DNA ends were deemed as passed, while invitrosomes containing a cutsite for the respective restriction enzyme within the nucleosomal DNA would normally be discarded are recovered. Using the alternate library’s passed invitrosomes, suspect invitrosomes were re-classified as recovered if a passed invitrosome from the other library could be found at the same position as the suspect invitrosome. The comparative analysis increases the sequencing data available for downstream analysis. (TIF) [file pone.0258737.s001.tif]

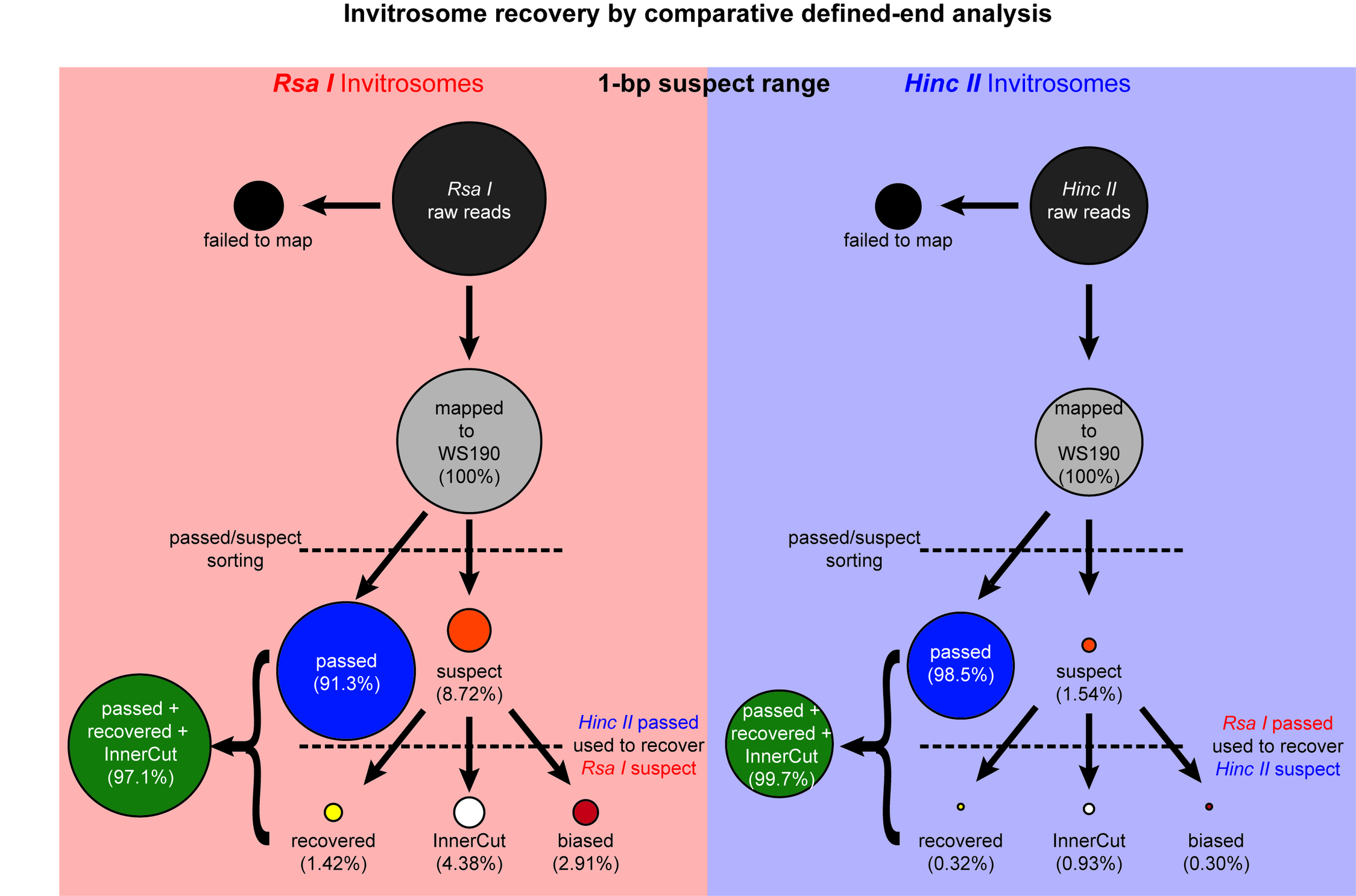

Supplement: S2 Fig — Visual depiction of the percentages of reads, and by extension invitrosomes, during the classification and re-classification method. Invitrosomes too close to DNA fragment ends (in this case within 1 base pair) were deemed suspect to formation on fragment ends due to end bias. Invitrosomes outside of 1 base pair from DNA ends were deemed as passed, while invitrosomes containing a cutsite for the respective restriction enzyme within the nucleosomal DNA would normally be discarded are recovered. Using the alternate library’s passed invitrosomes, suspect invitrosomes were re-classified as recovered if a passed invitrosome from the other library could be found at the same position as the suspect invitrosome. The comparative analysis increases the sequencing data available for downstream analysis. (TIF) [file pone.0258737.s002.tif]

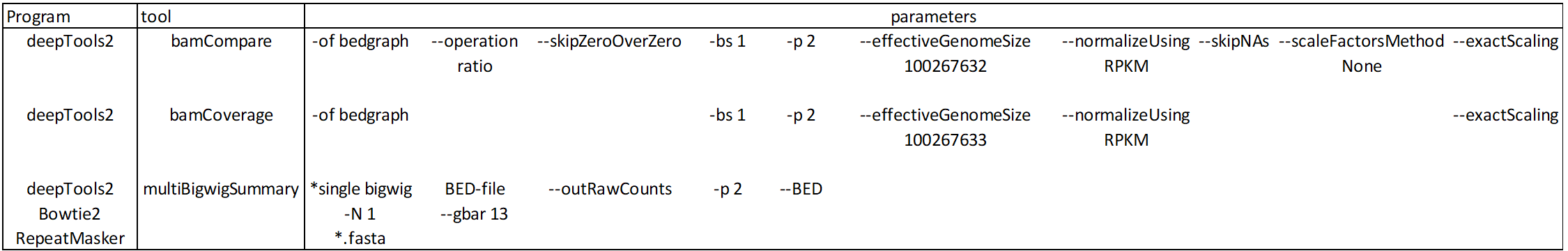

Supplement: S1 Table — Table of publicly available programs used in the analysis. All parameters for programs were set to default except as noted. (TIF) [file pone.0258737.s003.tif]
